# Body dissatisfaction among undergraduate medical students in the city of Juiz de Fora, Minas Gerais, Brazil

**DOI:** 10.31744/einstein_journal/2022AO6648

**Published:** 2022-04-28

**Authors:** Bruno Cassiano dos Santos, Dominique D’Alessandro Conte de Almeida, Nathália Vital Guilarducci, Rachel Rocha Pinheiro Machado

**Affiliations:** 1 Faculdade de Ciências Médicas e da Saúde de Juiz de Fora Juiz de Fora MG Brazil Faculdade de Ciências Médicas e da Saúde de Juiz de Fora, Juiz de Fora, MG, Brazil.

**Keywords:** Body image, Nutritional status, Students, medical, Feeding and eating disorders, Mental health

## Abstract

**Objective:**

To assess the level of body dissatisfaction among undergraduate medical students in the city of Juiz de Fora, Minas Gerais, Brazil.

**Methods:**

A cross-sectional study with 232 volunteers of both sexes at a private college. The Body Shape Questionnaire was used, which is a tool based on the sum of values that allow classifying body dissatisfaction according to the following scores: less than 111, if absence of body dissatisfaction; between 111 and 138, if mild body dissatisfaction; between 139 and 167, if moderate body dissatisfaction, and from 168, if severe body dissatisfaction. In addition, the self-reported body mass index and an assertion were used to assess the degree and perception (insight) of body dissatisfaction. For the statistical analysis, descriptive comparison, and binary logistic regression tests were performed.

**Results:**

The mean result of the Body Shape Questionnaire among women was 96.0±34.1 and among men, 76.7±24.7, with 26.3% of students with some level of dissatisfaction with self-image. Most participants (76.3%) wished to have a body mass index lower than the real one. Women (odds ratio of 5.7), overweight individuals (odds ratio of 6.1), and individuals with insight into their condition (odds ratio of 89.7) were more likely to be dissatisfied with the body image measured by the Body Shape Questionnaire.

**Conclusion:**

The search for a thin body among undergraduate medical students is a reality. In addition to overweight individuals, the female population has a significant level of body distortion, being recognized as the highest rate of body dissatisfaction in the sample surveyed.

## INTRODUCTION

Throughout history, the standards of beauty have changed according to the culture and the dictates of the wealthier social classes. Until the 18^th^ century, the most voluminous body was considered the most beautiful, since it represented all the wealth of the noble population, and, comparatively, the thin body was common to the poorest brackets of society. Along the middle of the same century, this model gradually underwent changes, based on European standards, which started to consider the thin body as the ideal to be achieved.^(1-4)^

With this new paradigm of the cult of thinness, everything that does not satisfy it is considered inadequate and unhealthy.^(1)^Although the metabolic problems that obesity can cause are well known, there is a notable stigmatization of the individual with a body that escapes the thin stereotype, inducing them to seek ways to reach this standard, considered ideal - even if, to do so, it is necessary to put mental and organic health at risk, with adherence to restrictive and low calorie diets, abuse of anorexic drugs and purgative acts, among other risky behaviors.^(5-7)^

Moreover, apparently, the search for a standardized body does not affect all individuals equally. The young female population seems to suffer more, since the dissemination of this content by the media and culture tends to target this audience. Some studies show that women have a much higher degree of distortion in perception of body self-image than men, and are more affected by eating disorders, such as anorexia and bulimia.^(5-10)^What is surprising is that some evidence, although scarce, suggests the population of health sciences students is even more affected by these phenomena.^(11,12)^

## OBJECTIVE

To assess the level of body dissatisfaction among undergraduate medical students in the city of Juiz de Fora, Minas Gerais, Brazil.

## METHODS

### Sample selection

For the sample size, the number of individuals enrolled in the medical course in the research period (n=759) was taken into consideration, with prevalence of the unknown problem (50%) and 95% confidence interval, with absolute precision of 7 points more or less, obtaining a minimum sample of 156 undergraduate students - already corrected for finite samples.^(13)^ Based on this value, the necessary population of men and women was found, based on the proportionality of individuals enrolled in the course (33.8% were men, and 66.2% were women). At the end of data collection, the study had more students than the minimum expected, totaling 56 men, and 176 women.

To participate in the sample, it was necessary to be enrolled between the first and the 12^th^ period of the medical course and be over 18 years old.

### Instruments and procedures

#### Body Shape Questionnaire

The translated Body Shape Questionnaire (BSQ), adapted and validated for a population of Brazilian undergraduate students, is a questionnaire with 34 self-completed items, designed to measure the concern with body shape and weight in the last 4 weeks. Each question has six answer options, ranging from one (never) to six (always). At the end, the values found are summed up, and a score is obtained that reflects the degree of body dissatisfaction. Values lower than 111 indicate absence of dissatisfaction; between 111 and 138, mild dissatisfaction; between 139 and 167, moderate dissatisfaction, and values over 168 reveal severe dissatisfaction with one’s body.^(14)^

#### Body mass index

Another tool used to evaluate the degree of body dissatisfaction was the body mass index (BMI). First, the volunteers referred their weight and height and, later, they indicated the weight and height they wished to present at the time of the study. The World Health Organization (WHO) classification was used to define the nutritional status based on the BMI, calculated with the two variables acquired. Thus, values below 18.5 indicated thinness; between 18.5 and 24.9, normal weight; between 25 and 29.9, overweight, and BMI over 30 indicated obesity.^(15)^ Thus, the analysis of this variable was performed assuming that actual BMI equal to desired BMI reflects absence of body dissatisfaction. Actual BMI higher than the desired BMI reflects body dissatisfaction with desire to decrease body volume; actual BMI lower than desired BMI also reflects body dissatisfaction, but with desire to increase body volume. The literature points out that when it is impossible to measure weight and height, or in population studies in which it is necessary to reduce costs, or even speed up the processes, self-reported data present a great correlation with reality.^(16-18)^

## Insight on body dissatisfaction

At the end of the questionnaire, the following assertion was added: “Have you been dissatisfied with your body in the last 4 weeks?”. This question, formulated by the authors of this study, aimed to analyze the perception (insight) of the volunteers regarding the level of body dissatisfaction, as assessed by the BSQ. The answer options were the same as in the BSQ: never, rarely, sometimes, frequently, very frequently, and always. To interpret this variable, we chose to dichotomize it, with “never,” “rarely,” and “sometimes” being related to the absence of body dissatisfaction, and “frequently”, “very frequently”, and “always” being considered as the presence of body dissatisfaction.

## Microsoft forms

All content was completed online through the Microsoft Forms platform contained in the Office 365^®^ package. The questionnaire was e-mailed to all students enrolled in the medical school. To access it, the volunteer had to be linked to a personal Microsoft institutional account, to avoid duplicate answers and information from populations other than the one selected to compose the scope of the study.

## Statistical analysis

For data organization and statistical analysis, the software IBM (SPSS), version 20, was used, with a significance level of 5% (α=0.05) for all tests. Initially, the descriptive evaluation of all quantitative data as minimum, maximum, mean, and standard deviation (SD) was performed, in addition to simple and relative frequencies.

To perform the statistical tests, the qualitative variables were considered instead of their numerical counterparts. Thus, non-parametric tests were used to analyze the data.

The relation between the BSQ results and sex was evaluated using Pearson’s χ^2^ test. The Kruskal-Wallis test was used to relate the BSQ to the course periods and the participants’ actual BMI. This same test was used to verify the relation between the actual BMI and the desired BMI of each study volunteer.

Finally, a binary logistic regression was performed to predict the odds of an individual’s body dissatisfaction (measured by the BSQ) as a function of nutritional status, sex, and the answer to the question about insight into body condition, added at the end of the questionnaire by the authors of this study.

To meet the requirements of the χ^2^ test, the BSQ was divided into two categories: satisfied (scores below 111) and dissatisfied (scores above 110). In addition to the dichotomization of the BSQ, to perform the binary logistic regression, it was necessary to group the nutritional status results into thinness-normal weight (BMI <25) and overweight-obesity (BMI ≥25).

The course periods were grouped into three cycles: from the 1^st^ to the 4^th^ (basic cycle), from the 5^th^ to the 8^th^ (clinical cycle), and from the 9^th^ to the 12^th^ period (internship), to enable the evaluation of this variable by the Kruskal-Wallis test.

## Ethical aspects

This study has an observational, cross-sectional, and individualized design, and was conducted in the city of Juiz de Fora, between June and September 2020, according to resolution 466/12, of the National Health Council, after approval by the Research Ethics Committee of the *Faculdade de Ciências Médicas e da Saúde de Juiz de Fora*, under # 3.958.312 and CAAE: 30257020.6.0000.5103.

## RESULTS

This study had 232 volunteers, with age ranging from 18 to 44 years, a mean±SD of 22.4±3.3 years for females, and 23.6±5.2 years for males. Of all volunteers, 93 (40.1%) were in the basic cycle, 106 (45.7%) in the clinical cycle, and 33 (14.2%) in internship.

The BSQ score ranged from 39 to 181; the mean score for women was 96.0±34.1, while for men it was 76.7±24.7. Of the 232 participants, 171 (73.7%) had no body distortion, and 61 (26.3%) had some level of dissatisfaction with self-image.

**Figure 1:**
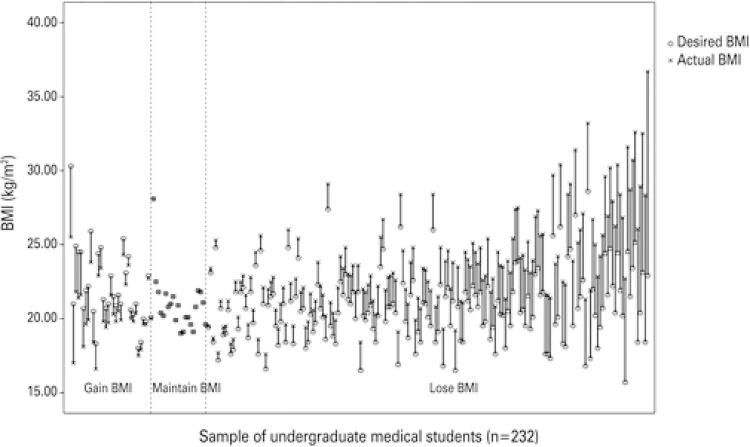
Comparison between actual and desired body mass index by undergraduate medical students in the city of Juiz de Fora

Of the dissatisfied, 54 were women (30.7%), and seven were men (12.5%), so there was a significant difference between the BSQ results and the sex of participants (p=0.007). In the comparison between the BSQ results and the course cycles, no significant difference was found, as shown on table 1. The mean actual BMI was 22.3±3.0kg/m^2^ for women and 24.9±3.5kg/m^2^ for men. The mean desired BMI was 20.3±1.9kg/m^2^and 23.1±2.6kg/m^2^ for women and men, respectively (Figure 1). Hence, there was a significant difference between actual and desired BMI (p<0.001), with a trend to have lower values (Table 2).

**Table 1:** Comparison of Body Shape Questionnaire results with sex, body mass index, and course cycle in a sample of medical students

	BSQ					p value
	
	Total	Absent body dissatisfaction	Mild body dissatisfaction	Moderate body dissatisfaction	Severe body dissatisfaction	
Sex						
Male	56 (24.1)	49 (21.1)	6 (2.6)	1 (0.4)	0	
Female	176 (75.9)	122 (52.6)	29 (12.5)	23 (9.9)	2 (0.9)	
Total	232 (100.0)	171 (73.7)	35 (15.1)	24 (10.3)	2 (0.9)	
Actual BMI, kg/m^2^						0.007*
Thinness	7 (3.0)	7 (3.0)	0	0	0	
Normal weight	175 (75.4)	136 (58.6)	24 (10.4)	15 (6.4)	0	
Overweight	40 (17.2)	21 (9.0)	10 (4.3)	7 (3.0)	2 (0.9)	
Obesity	10 (4.3)	7 (3.0)	1 (0.4)	2 (0.9)	0	
Total	232 (100.0)	171 (73.7)	35 (15.1)	24 (10.3)	2 (0.9)	
Cycle, study periods						
Basic	93 (40.1)	68 (29.3)	18 (7.8)	7 (3.0)	0	
Clinical	106 (45.7)	79 (34.0)	14 (6.0)	11 (4.7)	2 (0.9)	0.94*
Internship	33 (14.2)	24 (10.4)	3 (1.3)	6 (2.6)	0	
Total	232 (100.0)	171 (73.7)	35 (15.1)	24 (10.3)	2 (0.9)	0.002^†^

Results expressed as n (%).

* Level of significance - Pearson’s χ^2^ test (c^2^); ^†^ level of significance - Kruskal-Wallis test.

BSQ: Body Shape Questionnaire; BMI: body mass index.

BMI: body mass index.

**Table 2:** Relation between the actual body mass index and the desired body mass index of undergraduate medical students

Actual BMI (kg/m^2^)	Desired BMI (kg/m^2^)					p value

	Total	Thinness	Normal weight	Overweight	Obesity	
Thinness	7 (3.0)	4 (1.7)	3 (1.3)	0	0	
Normal weight	175 (75.4)	29 (12.5)	144 (62.1)	2 (0.9)	0	
Overweight	40 (17.2)	2 (0.9)	33 (14.2)	4 (1.7)	1 (0.4)	0.001*
Obesity	10 (4.3)	0	6 (2.6)	4 (1.7)	0	
Total	232 (100.0)	35 (15.1)	186 (80.2)	10 (4.3)	1 (0.4)	

Results expressed as n (%).

* Level of significance - Kruskal-Wallis test.

BMI: body mass index.

Only 22 (9.5%) individuals were satisfied with their BMI. Of the 210 dissatisfied, 33 (14.2%) showed a desire to gain kg/m^2^, while the other 177 (76.3%) individuals expressed a desire to lose BMI points. The mean difference between desired BMI and actual BMI was -2.0±2.5 kg/m^2^.

When analyzing the BSQ results in relation to the nutritional status of the participants, a significant difference was found (p=0.002). It was noted that individuals classified as overweight were those who had the highest level of body dissatisfaction measured by the BSQ, while thin and obese individuals had no significant variation in the BSQ scores when compared to the normal weight group (p>0.05). Moreover, the group of individuals who showed interest in reducing BMI showed a higher mean BSQ, when compared to the groups of individuals who showed interest in maintaining or gaining kg/m^2^, but with no significant difference (Table 3).

**Table 3:** Relation between Body Shape Questionnaire scores with body mass index and body dissatisfaction in undergraduate medical students

BSQ	Mean	SD	p value
Actual BMI			0.002*
Thinness	65.1	14.7	
Normal weight	87.6	31.3	
Overweight	109.6	35.5	
Obesity	103.2	31.8	
Dissatisfaction with BMI			
Gain kg/m^2^, n=33	79.5	27.5	
Maintain BMI, n=22	73.5	20.2	0.32*
Lose kg/m^2^, n=177	95.8	34.1	

* Significance level - Kruskal-Wallis test.

BSQ: Body Shape Questionnaire; BMI: body mass index; SD: standard deviation.

Regarding insight into one’s dissatisfaction with self-image, 106 (45.7%) individuals showed no body dissatisfaction, while 126 (54.3%) did show body dissatisfaction.

The binary logistic regression model (Table 4) showed that overweight-obese participants were significantly more likely to present with body dissatisfaction, measured by the BSQ, than those who were classified as thin and normal weight (odds ratio of 6.1; p=0.001). This was also verified with the variable sex, and women were more likely to be dissatisfied (odds ratio of 5.7; p=0.007). Finally, individuals who showed body dissatisfaction with the question about insight into their condition were also more likely to be dissatisfied with the assessment performed by the BSQ (odds ratio of 89.7; p<0.001).

**Table 4:** Binary logistic regression model for checking body dissatisfaction measured by the Body Shape Questionnaire relative to nutritional status, sex, and insight of the research volunteers

Variables	β	SE	Wald	DF	OR	95%CI	p value
Overweight-obesity	1.815	0.55	10.9	1.0	6.1	2.1-18.0	0.001
Women	1.740	0.64	7.3	1.0	5.7	1.6-20.1	0.007
Insight	4.497	1.03	18.9	1.0	89.7	11.8-680.0	0.001

SE: standard error; DF: degrees of freedom; OR: odds ratio; 95%CI: 95% confidence interval.

## DISCUSSION

The mean overall BSQ was 91.3, higher than in all other studies that have attempted to assess the level of body dissatisfaction among male and female undergraduate medical students (74.2; 73.3; 71.9; 75.0).^(14,19,20,21)^

Since the present study was carried out in a context of coronavirus 2019 disease (COVID-19) pandemic, it is relevant to consider that factors related to this scenario may have influenced the results obtained, as a bias, increasing the level of body dissatisfaction among students. Such a hypothesis is corroborated by Castro et al.,^(22)^who, in a recent study, investigated the level of body dissatisfaction during this pandemic and presented variables, such as confinement, reduced physical exercise, and increased unhealthy diets, as intensifiers of body dissatisfaction. In another study, Swami et al.^(23)^verified increased stress and anxiety, generated by social isolation during the pandemic, were also related to greater body dissatisfaction.

In the validation study of the BSQ, Di Pietro et al.^(14)^ found 86.6% of individuals who presented with body satisfaction, and there were no volunteers with severe body dissatisfaction. In the present study, only 73.7% of participants were satisfied, with two volunteers classified as having severe body dissatisfaction, showing this problem is still a reality and possibly has worsened over the years.

Although women’s BSQ results were significantly worse than men’s, as described in other studies involving health sciences students,^(14,19,20,24-27)^ women’s means were similar to those already described, while men’s were considerably higher than those found in the literature.^(14,19,25,27)^This shows that the level of body dissatisfaction may be increasing among men, even though it still affects women more.

In the study, it was found that undergraduate medical students are not satisfied with their BMI and tend to desire a slimmer than their real silhouette. In this sample, only 9.5% of participants were satisfied with the relation between their weight and height. This finding is reinforced by what has already been described by Bosi et al.,^(28)^ in a cohort of undergraduate medical students in the city of Rio de Janeiro, who found 6.6% satisfaction with BMI among the participants. Moreover, in this study, most students would like to lose, on average, 2.0kg/m^2^ in BMI, a value similar to that of other studies evaluating undergraduate nutrition (1.5kg/m^2^)^(29)^ and medical students (2.7kg/m^2^ in the group of women).^(14)^

It was also found that 35 students wished to be thin (BMI <18.5kg/m^2^), 31 of whom did not belong to this stratum, and therefore four were thin and wished to remain as such. Furthermore, only 11 students would have liked to be in the overweight category (BMI ≥25.0kg/m^2^), while in reality, 50 volunteers were part of this group. This shows that the number of individuals who wished to be classified as thin by BMI was considerably higher than those who wished to be in the overweight-obese category, highlighting how much thinness is desired.

Some studies with undergraduate health sciences students have shown that high BMI values tend to predict a higher level of body dissatisfaction.^(24,26,30-35)^ This result was also found in this study, and the individuals classified as overweight were the most dissatisfied with their body image, including two volunteers with severe body dissatisfaction. Kakeshita et al.,^(34)^in their study with undergraduate students in the city of Ribeirão Preto (SP), also showed overweight volunteers were the most dissatisfied, but this finding differs from that of Kesller al.,^(36)^ who found the highest BSQ means in normal weight individuals.

Another evidence that shows greater severity of the problem is that 22.3% of normal weight individuals had some degree of body dissatisfaction (BSQ >110). This percentage was lower than that found in other studies,^(36-38)^ but even so, it represents a large portion of individuals who, even within the expected BMI values, do not feel satisfied with their body image.

Although medical students are considerably dissatisfied with their body self-image, no significant difference was found between body dissatisfaction and the cycles in which the participants were studying at the time of the survey. This finding is similar to other studies,^(19,21)^showing the problem tends not to change throughout medical training.

With the binary logistic regression model, we found a 6.1-fold increase in overweight students with body dissatisfaction. This finding is higher than that reported in other studies^(21,24,35,37)^ and similar to that reported among undergraduate nutrition students in the city of Ouro Preto (MG).^(29)^ This tendency to be dissatisfied with the body, considered overweight, reinforces the stigmatization of this pattern, which is not restricted to health issues. Women were also more likely to present body dissatisfaction with a proportion 5.7 times higher than that of men. This chance of risk was higher than the one found by Silva et al.,^(39)^ (odds ratio of 2.2; p<0.001) among undergraduate physical education students in Southern Brazil and lower than that reported by Regis et al.,^(21)^ among undergraduate medical students in Botucatu (odds ratio of 13.5; p<0.05). This variation is probably due to cultural beliefs, social media, diets, level of physical activity, and other factors that make up the body image construct,^(19,35,40)^ but reveal that, in any case, women are the most dissatisfied with their own bodies.

This is the first study that brings an additional investigative question, designed by the authors, which aimed to evaluate the perception (insight) of body dissatisfaction by the volunteers. With the analysis of the values found in the binary logistic regression, this statement seems to predict more chances of body dissatisfaction measured by the BSQ (odds ratio of 89.7; 95%CI: 11.8-680.0; p<0.001) when the individuals answer that they were often or always dissatisfied with their own body in the previous month. Thus, this question seems to be useful to help health professionals at times when the BSQ or other scales that aim to measure body dissatisfaction cannot be applied due to time constraints, since it takes about 10 minutes to answer the questionnaire. This is a preliminary conclusion that needs to be reproduced and validated in future studies.

One of the limitations of this study concerns its methodological transversality, making cause and effect relations impossible. There was no control or evaluation of other variables, such as ethnicity, income, physical activity, diet, and other factors that contribute to the formation of the body image construct. Another limitation was the participants’ self-reported anthropometric measurements, which, although valid, are not necessarily accurate. It is also noteworthy the weight/height relation that generates the BMI has, by nature, the limitation of not differentiating lean mass from fat mass, so that some values may be under- or overestimated.^(41)^ Finally, the results expressed in this study cannot be generalized to the entire population of undergraduate medical students, since the study was carried out in only one college.

Although there are limitations, this study is relevant because it gives a dimension of body dissatisfaction in undergraduate medical students of a private college, and is similar to findings already described in the literature. This is essential, since the physician is one of the professionals involved in the management of people with body image disorders, and it is crucial for good training and assistance to be aware and informed about these problems.

In the literature, studies that evaluate the body image of undergraduate medical students are scarce. In this sense, the reproduction of this type of work in other educational organizations is essential for comparisons that are more reliable with the findings already published on the subject. Furthermore, longitudinal methodological designs are essential for the development of causal relations among the variables that form the body image construct. Finally, we suggest the reproduction and validation of the question about the insight of individuals regarding body dissatisfaction in future studies.

## CONCLUSION

Being dissatisfied with body image is a reality among undergraduate medical students who tend to value the thin body over other standards. Individuals with insight into their body dissatisfaction are more likely to have worse Body Shape Questionnaire scores. It was also observed that women and other overweight participants are significantly more dissatisfied than men and those who are not overweight. However, the dissatisfaction presented by male students and by normal weight students is equally worrisome, and shows a problematic panorama regarding the beauty ideal of these individuals.
